# Effects of Process Conditions on the Mechanical Behavior of Aluminium Wrought Alloy EN AW-2219 (AlCu6Mn) Additively Manufactured by Laser Beam Melting in Powder Bed

**DOI:** 10.3390/mi8010023

**Published:** 2017-01-16

**Authors:** Michael Cornelius Hermann Karg, Bhrigu Ahuja, Sebastian Wiesenmayer, Sergey Vyacheslavovich Kuryntsev, Michael Schmidt

**Affiliations:** 1 Institute of Photonic Technologies (LPT), Friedrich-Alexander-Universität Erlangen-Nürnberg FAU, Konrad-Zuse-Straße 3/5, 91052 Erlangen, Germany; Bhrigu.Ahuja@lpt.uni-erlangen.de (B.A.); Sebastian.Wiesenmayer@fau.de (S.W.); Michael.Schmidt@lpt.uni-erlangen.de (M.S.); 2 Collaborative Research Center 814—Additive Manufacturing (CRC 814), Am Weichselgarten 9, 91058 Erlangen-Tennenlohe, Germany; 3 Erlangen Graduate School in Advanced Optical Technologies (SAOT), Paul-Gordan-Straße 6, 91052 Erlangen, Germany; 4 Department of Laser Technologies, Kazan National Research Technical University, K. Marx Str. 10, 420111 Kazan, Russia; Kuryntsev16@mail.ru

**Keywords:** additive manufacturing, 3D printing, powder bed fusion, aluminium copper wrought alloy EN AW-2219, AlCu6Mn, tensile test, Selective Laser Melting™

## Abstract

Additive manufacturing is especially suitable for complex-shaped 3D parts with integrated and optimized functionality realized by filigree geometries. Such designs benefit from low safety factors in mechanical layout. This demands ductile materials that reduce stress peaks by predictable plastic deformation instead of failure. Al–Cu wrought alloys are established materials meeting this requirement. Additionally, they provide high specific strengths. As the designation “Wrought Alloys” implies, they are intended for manufacturing by hot or cold working. When cast or welded, they are prone to solidification cracks. Al–Si fillers can alleviate this, but impair ductility. Being closely related to welding, Laser Beam Melting in Powder Bed (LBM) of Al–Cu wrought alloys like EN AW-2219 can be considered challenging. In LBM of aluminium alloys, only easily-weldable Al–Si casting alloys have succeeded commercially today. This article discusses the influences of boundary conditions during LBM of EN AW-2219 on sample porosity and tensile test results, supported by metallographic microsections and fractography. Load direction was varied relative to LBM build-up direction. T6 heat treatment was applied to half of the samples. Pronounced anisotropy was observed. Remarkably, elongation at break of T6 specimens loaded along the build-up direction exceeded the values from literature for conventionally manufactured EN AW-2219 by a factor of two.

## 1. Introduction

### 1.1. Terminology of Additive Manufacturing Technology

**Figure 1:**
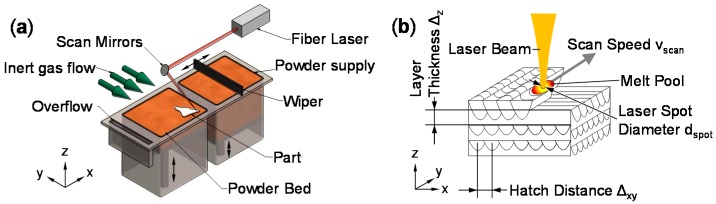
(**a**) Basic machine setup of Laser Beam Melting in Powder Bed (LBM); (**b**) 3D build-up from overlapping weld tracks.

This paper is dedicated to additive manufacturing from a metal powder bed without binder using a laser beam. ISO/ASTM F52900 and ISO 17296 define a higher-level category “powder bed fusion” including other technologies that employ incoherent radiation, laser or electron beams to process polymers, ceramics or metals with or without binder [1,2]. In this paper, the precise technology of interest is referred to as Laser Beam Melting in Powder Bed (LBM) in the style of VDI 3405 [3]. Figure 1a shows the machine principle and Figure 1b the process variables. An established trademark for LBM (among others) is, for example, Selective Laser Melting™ (SLM™) [4].

### 1.2. Terminology of Aluminium Alloys

European Standard EN 12258-1 defines an Al wrought alloy as an “alloy primarily intended for the production of wrought products by hot and/or cold working” and likewise for casting alloys [5]. AW stands for “aluminium wrought” in alloy designations [6]. Alloys are defined by chemical compositions [7,8,9]. “Wrought” and “casting” are part of Al alloy designations. In this paper, processing is stated as either by LBM or conventionally; the latter meaning by working or casting, depending on the alloy. Table 1 shows the composition of EN AW-2219 used for LBM experiments.

### 1.3. Motivation

Industrial use of and research on LBM have increased rapidly in recent years [4], but the material spectrum is still limited. LBM shows great potential for functionally optimized and light-weight designs. For such applications, conventionally manufactured Al wrought alloys are established due to high strength-to-weight ratios, predictable mechanics that are adjustable by heat treatment and the ability to avoid sudden failure by plastic deformation [10,11].

The most common Al alloy in LBM today is AlSi10Mg, among other Al–Si casting alloys [12,13,14,15]. AlSi10Mg is also used as filler material for welding Al–Cu wrought alloys [16]. Ultimate tensile strength (UTS) of 320–360 MPa and elongation at break (E) of 2%–8% of LBM AlSi10Mg T6 were reported [13]. According to [13], elongation at break is higher orthogonal to the build-up direction than parallel to it. This anisotropy was not reported in [17] based on a round robin with machine manufacturers and academic institutions. Established laser power for LBM of Al alloys is 400 W; research experiments with 1000 W have been published [18]. Al–Mg–Sc alloys have been continuously researched in LBM [19,20,21]. Industrial use might be impeded by high limited global annual Sc production of only 10–15 t [22]. LBM of very high strength Al–Zn alloys encountered issues of cracking and alloy changes by evaporation of Zn [23,24]. EN AW-7075 plus 4 wt % Si was crack-free after LBM with relative density ρ_rel_ = 98.9%, but Zn content and tensile test results of this Al–Si–Zn alloy were not published [14,25]. Loss of Zn in LBM of Al–Zn–Mg–Cu was reported in [26] and micrograph areas <0.2 mm were shown, but information about cracks, densities and tensile test results were lacking. LBM of 6xxx series Al–Mg–Si yielded cracks unsuitable for high mechanical performance [27,28].

LBM of EN AW-2022, EN AW-2024, EN AW-2618A and EN AW-2219 with ρ_rel_ > 99.9% [29,30] and results from tensile testing of EN AW-2618A [31] were published by the authors. Others followed to publish on LBM of 2xxx series Al–Cu wrought alloys [32,33].

Conventionally manufactured EN AW-2219 reaches 414 MPa ultimate tensile strength, 10% elongation at break and performs well at elevated temperatures [34].

The goal of this contribution is to investigate the mechanical properties of EN AW-2219 manufactured by LBM under consideration of heat treatment and build orientation relative to load.

## 2. Materials and Methods

### 2.1. Prealloyed Argon Atomized Powder with Chemical Composition of EN AW-2219

**Figure 2:**
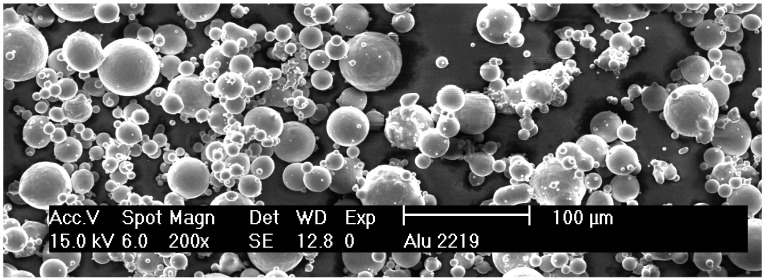
Scanning electron microscope (SEM) image of argon atomized and sieved EN AW-2219 powder.

Powder with the chemical composition of EN AW-2219 shown in Table 1 had been atomized with Ar by TLS (TLS Technik GmbH & Co Spezialpulver KG, Bitterfeld-Wolfen, Germany). It was vibration sieved at Institute of Photonic Technologies (LPT, Erlangen, Germany) under Ar between 20 and 63 µm mesh width. Scanning electron microscopy (SEM) on a Zeiss Merlin (Carl Zeiss Microscopy GmbH, Jena, Germany) in Figure 2 shows many remaining particles <20 µm.

### 2.2. Tensile Specimen Geometry and Build-Up Orientation

**Figure 3:**
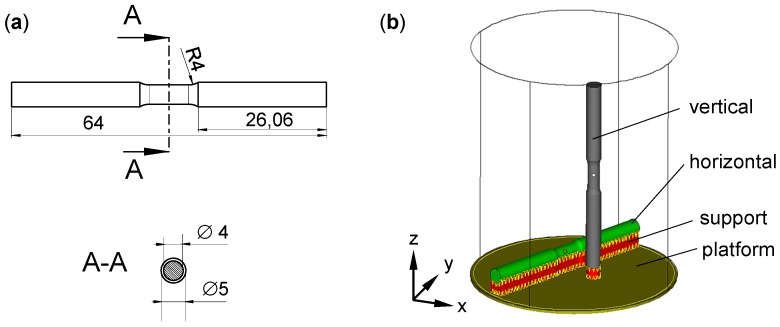
(**a**) Tensile specimen; testing area 8 mm long and 4 mm diameter, all dimensions in mm; (**b**) orientation of tensile specimens relative to build chamber of LBM machine.

The geometry of tensile specimens as shown in Figure 3a was chosen according to best practice. The overall length was 64 mm and the cross section was circular. The diameter of the testing area was 4 mm, that of clamping zones 5 mm, with a 4-mm radius as transition. The length of the testing area was 8 mm. The tensile samples were built in two orientations: vertical and horizontal, as shown in Figure 3b. The horizontal ones were tensile tested in parallel to the LBM layers, the vertical ones in parallel to the build-up direction. Twelve specimens were built in total, providing three identical samples of each combination of two orientations and two heat treatment states (T6 and as-built).

### 2.3. LBM Process

Tensile specimens were built on an SLM 50 LBM machine from ReaLizer GmbH (Borchen, Germany) with a single mode Yb fiber laser from IPG Photonics Corp. (Oxford, MA, USA) with 1070 nm wavelength and maximum output power of 100 W in continuous wave mode. LBM parameters as listed in Table 2 had been previously developed and published [30]. The cylindrical build envelope is 90 mm high with 70 mm diameter. Platforms were of AlMg3 and roughened by sand blasting for more homogeneous coating of the first powder layer and better welding connection to the supports. Scanning was meandering (two neighbouring scan vectors in a layer always pointed in opposite directions) and alternating (90° rotation of vectors around the vertical *z*-axis between layers). No stripes nor chessboard patterns were used. Scan speed of SLM 50 was set indirectly by point distance of 5 µm and exposure time of 40 µs. Actual scan speed *v*_scan_ of laser spot on powder bed had been determined with a high-speed camera Phantom V1210 (Vision Research, Wayne, NJ, USA) to be 157.4 mm/s. Supports were designed in Magics 17.11 (Materialise NV, Leuven, Belgium).

### 2.4. Post-LBM Processing of Samples

Half the samples were T6 heat treated. Solution annealing for 10 h at 530 °C in a chamber furnace Nabertherm N11/HR was followed by quenching in ice-water for repeatable temperature of 0 °C, artificial aging at 190 °C for 18 h and air cooling. After manual removal of platform and supports, all samples were shot peened with glass beads in an IEPCO Peenmatic 550 blasting cabin (IEPCO AG, Leuggern, Switzerland).

### 2.5. Metallography and Fractography

For metallographic analysis, samples of cubes and broken tensile specimens were embedded in epoxy resin, ground and polished with subsequently finer grain size down to 1 µm. Relative density ρ_rel_ by image analysis was calculated as quotient of black to white pixels. Bohner’s etchant revealed grain structure. Fracture surfaces were imaged via reflected light microscopy and SEM.

### 2.6. Mechanical Characterization

Tensile tests were conducted at room temperature according to ISO 6892-1 on a Zwick/Roell Z100 [35]. Stress and strain were recorded after reaching a pre-load of 1 MPa, ramped up with 1 mm/min. Until reaching yield strength, stress rate was constantly 20 MPa/s. Between yield strength and break, strain rate was constantly 0.008 s^−1^. Elongation was taken from the displacement of machine beams. Vickers micro-hardness HV 0.05 was measured on a Fischerscope H100VP (Helmut Fischer GmbH, Sindelfingen, Germany) according to ISO 6507-1 [36]. A force of 500 mN was ramped up over 20 s, kept for 5 s and ramped down over 20 s. Nine indentations with 2 mm distance were made per polished cube.

## 3. Experimental Results and Discussion

### 3.1. Metallography

**Figure 4:**
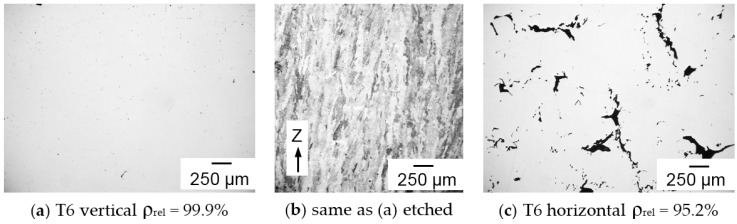
Polished cross sections: (**a**) vertical sample: very small round pores (black); (**b**) the same sample as in (a), but etched—directional grain structure; (**c**) horizontal sample—large irregular voids (black).

**Figure 5:**
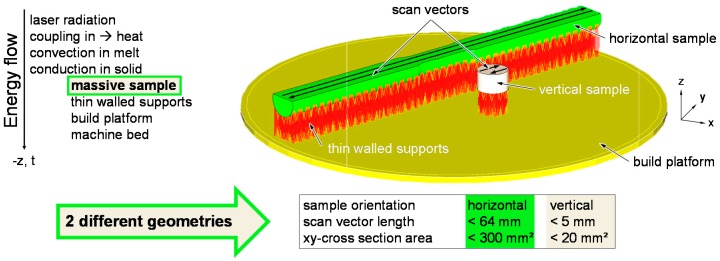
Geometry effects: scan vector length and cross-sectional area for vertical heat abduction.

Image analysis of polished cross-sections yielded a relative density ρ_rel_ = 99.9% for the vertical specimen in Figure 4a compared to ρ_rel_ = 95.2% for the horizontal one in Figure 4c. A typical threshold for scrap LBM parts is ρ_rel_ = 99.5% [3]. The porosity of horizontal samples was unacceptably high. Voids in horizontal samples had irregular shapes, indicating an origin from incomplete melting. Since all else was kept constant, the different geometry of vertical and horizontal samples illustrated in Figure 5 should explain the different ρ_rel_. The low laser power of 100 W and the high heat conductivity and capacity of Al might have increased the sensitivity of the LBM process to different geometries.

All samples showed a pronounced directional grain growth along the build-up direction. An obvious reason for this is the mostly vertical temperature gradient during solidification. Before and after T6 heat treatment, columnar grains showed in etched micrographs as in Figure 4b. The surrounding powder is approximately a thermal insulator. The majority of heat input from irradiation must dissipate in the negative *z* direction through solid material into the build platform and frame of the LBM machine. Thin-walled supports have small cross-sectional areas and act as a resistance to heat abduction. The LBM parameters used (Table 2) yielded significantly lower ρ_rel_ if samples were built directly on the platform without supports using several Al alloys [29,30,37]. This shows a requirement to limit heat abduction. With the basic scan strategy used here, the scan vector length was much longer on average in horizontal samples, as illustrated in Figure 5. Because *v*_scan_ was constant, any fixed point in the processing plane had a longer time to cool down between two overlapping passes of the melt pool on average (Figure 1b), compared to vertical samples. A second contribution might be from larger cross-sectional area of horizontal samples. As in Figure 5, the main direction of heat transfer is along the negative *z*-axis. Heat is input by coupling laser radiation energy into the dynamic melt pool. Below, heat is conducted through the solidified Al alloy through supports and the build platform to the machine frame. The larger cross-sectional area of the horizontal samples offers lower resistance to heat conduction than the smaller one of vertical samples. This should speed up heat abduction. Horizontal samples might qualitatively be described as a parallel connection of resistances to heat conduction. In comparison, vertical tensile specimens with their smaller cross-sectional area parallel to the build platform and their larger *z*-height might be described as serial connections of heat flow resistances.

Longer and faster local cooling may have caused insufficient melting. LBM parameters were developed on 5 mm cubes [30] with scan vectors and cross sections similar to vertical tensile samples.

### 3.2. Fractography

**Figure 6:**
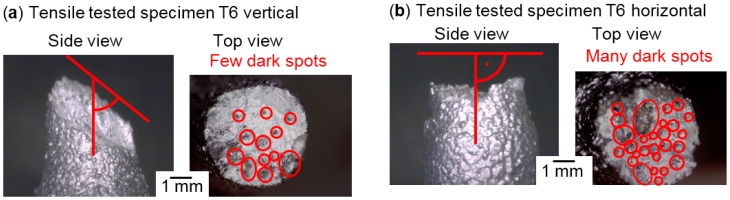
Side and top views in reflected light microscopy of fracture surfaces (**a**) inclined and (**b**) orthogonal to the pulling direction. For larger magnifications of dark spots, see Figure 7 and Figure 8.

**Figure 7:**
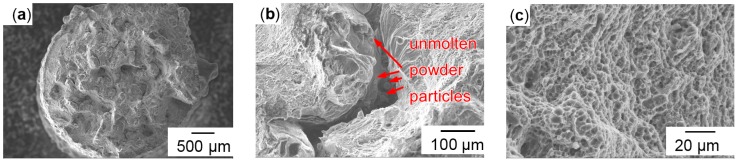
Horizontal non-heat-treated (**a**) overview; (**b**) unmolten particles; (**c**) fibrous structures.

The fracture surfaces of vertical specimens appeared more homogeneous then of horizontally built ones, as shown in Figure 6. Some dark and shiny spots were visible on the breaking surfaces, but fewer and smaller ones than on the horizontal surfaces in Figure 6b. Matte bright grey surfaces indicate ductile fracture, as does the inclination of the fracture surface to the pulling direction of roughly 45° in Figure 6a, which is the plane of maximum shear stresses in tensile tests. The horizontal specimens showed separation planes—however irregularly shaped—approximately orthogonal to the pulling direction. Many crescent-shaped defects are visible, as shown in Figure 7b. These vary in size from about 50 to 500 µm, and persisted through T6 heat treatment. Their size and rounded shape indicate an origin from drop-like melt formations that did not sufficiently connect with the subjacent material. Considering the pulling direction being parallel to the LBM layers, it seems plausible that these crescents are the bottom outlines of melt tracks, as illustrated in Figure 1b. The SEM image in Figure 7b shows spherical structures highlighted in red circles. These might be some of the smaller powder particles that had not been molten, considering the particle size distribution illustrated in Figure 2. Terrace-like edgy breaking surfaces visible in Figure 7a indicate that these crescent-shaped defects with unmolten particles highlighted red in Figure 7b might have initiated cracks with subsequent ductile fracture. Clues for ductile plastic deformation are the web-like fibrous structures in Figure 7c.

**Figure 8:**
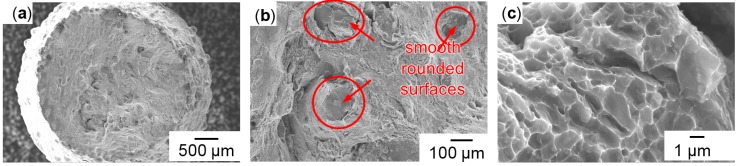
Vertical non-heat-treated (**a**) overview; (**b**) rounded surfaces; (**c**) fibrous structures.

**Figure 9:**
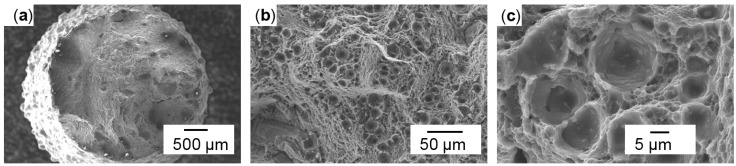
Vertical heat-treated (**a**) overview; (**b**) detail with rounded surfaces; (**c**) dimple structures.

**Figure 10:**
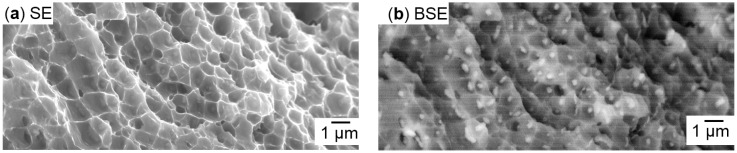
(**a**) Secondary electron (SE) and (**b**) back scattered electron (BSE) contrasts of the same region of the fracture surface of a non-heat-treated vertical sample reveal spots of different elements.

**Figure 11:**
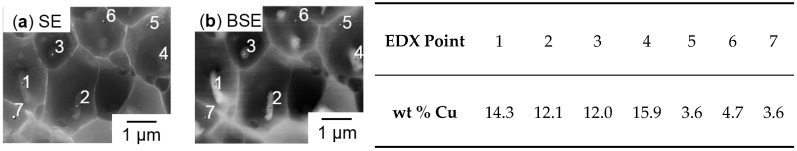
Non-heat-treated vertical specimen in (**a**) SE and (**b**) BSE contrast; energy dispersive X-ray spectroscopy (EDX) point measurements 1–4 showed higher Cu-contents in particles than in points 5–7 in the background.

The vertical samples in general appeared more homogeneous than the horizontal ones, as shown in Figure 8 as opposed to Figure 7. The vertical specimens showed more ductile fracture features, which increased after T6 heat treatment (as shown in Figure 9). The separation planes were more inclined to the sample axes, and the fracture surfaces were less irregular. Contrary to the crescent-like shapes found in horizontal samples, there were noticeable defects with smooth, curved surfaces in the vertical samples, highlighted red in Figure 8b. Such surface morphology is typically formed from melt solidification, indicating incomplete melt coalescence with the previously built underlying layer. The larger magnification in Figure 8c shows a fibrous honey-comb-like surface structure, indicating a fracture with more plastic deformation. In both heat treatment states (T6 and as-built), dimples around the size of 1–10 µm were found, as shown in Figure 9b,c. Small particles of deviating chemical constitution appeared in high magnification back scattered electron (BSE) contrast at the centres of dimples, as shown in Figure 10. Energy dispersive X-ray spectroscopy (EDX) in Figure 11 confirmed higher Cu content of these particles compared to background and nominal alloy composition in Table 1. The Cu-rich particles are probably intermetallic phases precipitated from the Al matrix.

### 3.3. Mechanical Characterization

The mean Vickers microhardness of LBM EN AW-2219 as-built of nine indentations was 94 HV0.05 ± 6.6 HV0.05. After T6, it increased to 147 HV0.05 ± 2.3 HV0.05, which can be attributed to precipitation hardening. Conventionally manufactured EN AW-2219 T62 reaches 130 HV according to [34], which is significantly lower. The state-of-the-art LBM aluminium casting alloy AlSi10Mg showed microhardness between 140 HV and 150 HV as-built [19] and between 80 HV and 120 HV after T6 heat treatment [13]. These values compiled in Table 3 illustrate the very different metallurgy of almost eutectic Al–Si casting alloy AlSi10Mg that is softened by T6 heat treatment.

**Figure 12:**
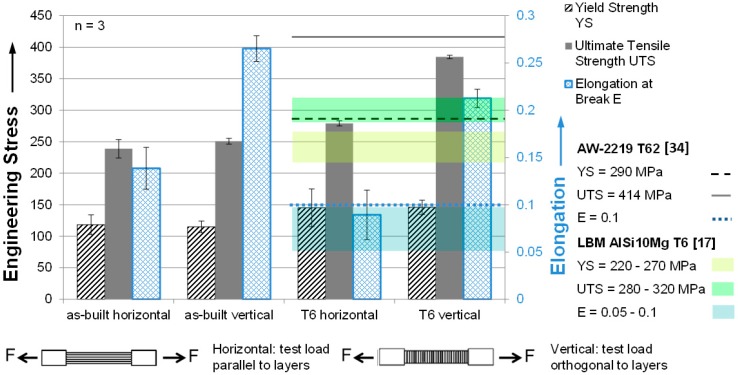
LBM AW-2219 tensile tests compared to literature values of conventionally manufactured EN AW-2219 T62 and LBM AlSi10Mg T6. No literature is known describing LBM EN AW-2219.

Yield strength (YS), ultimate tensile strength (UTS), and elongation at break (E) obtained from tensile tests are illustrated in Figure 12. Except for E, all characteristics benefit from T6 compared to the as-built state, as expected from a precipitation hardening alloy like EN AW-2219. Most remarkably, E of T6 vertical samples is more than two times as high as of conventionally processed EN AW-2219 T62. YS of all samples is far below values for conventionally manufactured EN AW-2219 from [34]. With 384 MPa, T6 vertical samples do reach 93% of the literature value for UTS of EN AW-2219. Comparing to the state-of-the-art LBM Al alloy AlSi10Mg [18], YS of LBM EN AW-2219 T6 was again much lower. UTS of horizontal T6 samples was only slightly below LBM AlSi10Mg T6, that of vertical T6 samples exceeded it by 65 MPa. Elongation at break of horizontal EN AW-2219 samples was within the range of LBM AlSi10Mg T6 and that of vertical samples more than twice as high.

Except for YS, characteristics were highly anisotropic after T6. Considering the unacceptably high porosity of the horizontal samples, this seems to be self-evident. In the as-built condition, YS and UTS were isotropic. Because of the poor horizontal sample quality, it seems not to be justified to discuss potential effects of the columnar grain structure, however anisotropic. Additional error sources must be taken into account. Only three identically prepared specimens of each variation were tested. The rough LBM surfaces were smoothed by glass bead blasting. This might have led to residual compressive stresses and thus slightly improved mechanical performance. On the other hand, the remaining roughness was still greater than that of conventionally machined samples, which should be detrimental to tensile testing performance. Rough samples might also more easily slip in the hydraulic clamping units of the testing machine. However, such slippage should result in irregularities in the stress–strain curves (e.g., exceptionally low slopes or repeated kinks). No such obvious irregularities could be found in the data.

Overall, results and their dependency on build orientation appeared to be very similar to those published in [31] for LBM of EN AW-2618A in a very similar experimental setup, supporting the interpretation that they are mainly caused by LBM process conditions.

## 4. Conclusions

The production of vertical tensile specimens with low porosity and without cracks from EN AW-2219 on an LBM machine of 100 W laser power is possible. However, process stability was found to be highly sensitive to geometry at identical volumetric energy density. Horizontal samples had roughly 5% porosity that was most probably caused by incomplete fusion. This is attributed to effectively longer local cooling time due to longer scan vectors and faster cooling rate due to larger cross-sectional areas. The sensitivity of the LBM results to these geometry effects might be raised by the combination of a material with high heat conductivity and capacity and the low laser power.

When loaded in build-up direction, elongation at break exceeded two times the literature values of conventionally processed EN AW-2219 T62 samples. As-built samples did not meet literature values for conventionally processed EN AW-2219 T62. Vertically built and T6 heat treated samples were not competitive in yield strength, but reached 93% of tensile strength. Columnar grains parallel to the build-up direction were distinct as-built and after T6. The porous horizontal samples showed inferior mechanical characteristics. Fracture surfaces appeared to be very heterogeneous, especially of the horizontal samples. Incompletely connected melt tracks were found in addition to indications of ductile fracture. Microhardness of T6 samples of 140 HV was higher than the 130 HV of conventionally processed EN AW-2219 T6 [34]. Overall, dependency of results on build orientation appeared similar to [31] with AW-2618A in a very similar experimental setup, supporting the interpretation they were mainly caused by geometric LBM process conditions.

For future experiments in LBM of EN AW-2219, scan strategies with limited vector length like stripes or chess board may enable fabrication of horizontal samples with competitive relative density above 99.5% and improved melt track connection. By testing and comparing samples of acceptable quality from both orientations, effects of columnar grain structure, interactions of long LBM jobs, post-build heat treatment and precipitations should be more accessible.

## Figures and Tables

**Table 1:** Composition of EN AW-2219 according to [7] in wt %; single numbers mean upper limits.

Cu	Mn	Ti	V	Zr	Zn	Mg	Si	Fe	Al
5.8–6.8	0.2–0.4	0.02–0.1	0.05–0.15	0.1–0.25	0.1	0.02	0.2	0.3	balance

**Table 2:** Overview of LBM parameters used to build tensile specimens.

*P*_Laser_	*v*_scan_	Δ*_xy_*	Δ*_z_*	*d*_spot_	*T*_platform_	Gas
100 W	157 mm/s	90 µm	30 µm	65 µm	200 °C	Ar

**Table 3:** Vickers micro-hardness HV0.05 of LBM EN AW-2219 in as-built and T6 condition compared to literature values of conventionally manufactured EN AW-2219 T62 and LBM AlSi10Mg.

**Alloy**	EN AW-2219	EN AW-2219	EN AW-2219	AlSi10Mg	AlSi10Mg
**Processed**	LBM	LBM	Conventional [34]	LBM [19]	LBM [13]
**Heat Treatment**	As-built	T6	T62	As-built	T6
**Mean**	94 HV0.05 *n* = 9	147 HV0.05 *n* = 9	130 HV	140–150 HV	80–120 HV
**Standard Deviation**	6.6 HV0.05 *n* = 9	2.3 HV0.05 *n* = 9	-	-	-
